# Healthcare-associated infections and antimicrobial use in Belgian nursing homes: results of three point prevalence surveys between 2010 and 2016

**DOI:** 10.1186/s13690-022-00818-1

**Published:** 2022-02-18

**Authors:** Katrien Latour, Boudewijn Catry, Brecht Devleesschauwer, Frank Buntinx, Jan De Lepeleire, Béatrice Jans

**Affiliations:** 1 Department of Epidemiology and Public Health, Sciensano, Brussels, Belgium; 2 Department of Public Health and Primary Care, KU Leuven, Leuven, Belgium; 3 Faculty of Medicine, Université libre de Bruxelles, Brussels, Belgium; 4 Department of Translational Physiology, Infectiology and Public Health, Ghent University, Merelbeke, Belgium; 5 Department of General Practice, Maastricht University, Maastricht, The Netherlands

**Keywords:** Long-term care, Nursing homes, Infection, Anti-bacterial agents, Infection control, Cross-sectional studies

## Abstract

**Background:**

Belgium monitors the burden of healthcare-associated infections (HAIs) and antimicrobial use in nursing homes (NHs) by participating in the European point prevalence surveys (PPSs) organised in long-term care facilities (HALT surveys). We present the main findings of the three national PPSs conducted in NHs participating in at least one of these surveys, and in a cohort that participated in all three consecutive surveys.

**Methods:**

All NHs were invited to voluntarily participate and conduct the survey on one single day in May-September 2010 (HALT-1), in April-May 2013 (HALT-2) or in September-November 2016 (HALT-3). Data were collected at institutional, ward and resident level. A detailed questionnaire had to be completed for all eligible (i.e. living full time in the facility since at least 24 h, present at 8:00 am and willing to participate) residents receiving at least one systemic antimicrobial agent and/or presenting at least one active HAI on the PPS day. The onset of signs/symptoms had to occur more than 48 h after the resident was (re-)admitted to the NH.

**Results:**

A total of 107, 87 and 158 NHs conducted the HALT-1, HALT-2 and HALT-3 survey, respectively. The median prevalence of residents with antimicrobial agent(s) increased from 4.3% (95% confidence interval (CI): 3.5-4.8%) in HALT-1 to 4.7% (95% CI: 3.5-6.5%) in HALT-2 and 5.0% (95% CI: 4.2-5.9%) in HALT-3. The median prevalence of residents with HAI(s) varied from 1.8% (95% CI: 1.4-2.7%) in HALT-1 to 3.2% (95% CI: 2.2-4.2%) in HALT-2 and 2.7% (95% CI: 2.1-3.4%) in HALT-3. Our post-hoc analysis on the cohort (n = 25 NHs) found similar trends. In all three surveys, respiratory tract infections were most frequently reported, followed by skin/wound infections in HALT-1 and urinary tract infections in HALT-2 and HALT-3. Antimicrobials were most commonly prescribed for the therapeutic treatment of an infection: 66.4% in HALT-1, 60.9% in HALT-2 and 64.1% in HALT-3. Uroprophylaxis accounted for 28.7%, 35.6% and 28.4% of all prescriptions, respectively.

**Conclusions:**

None withstanding the limitations peculiar to the study design, the PPSs enabled us to assess the occurrence of and to increase awareness for HAIs and rational antimicrobial use in NHs at both local and national level.

**Supplementary Information:**

The online version contains supplementary material available at 10.1186/s13690-022-00818-1.

## Background

Over the last decades, the profile of residents in long-term care facilities (LTCFs) changed profoundly. Under pressure of an ageing population, the growing burden of chronic diseases and the trend towards reduced length of stay in hospitals, more specialised care is being provided in these healthcare institutions that include among others nursing homes (NHs), LTCFs for mentally or physically disabled persons, psychiatric facilities and rehabilitation centres [1, 2]. By accumulating diverse risk factors for colonization with multidrug-resistant organisms (MDROs) associated with this more specialised care (e.g. long stay, device use, comorbid conditions, antimicrobial use), residence in a LTCF itself has become an independent risk factor for the acquisition of antibiotic-resistant organisms [3].

Similar to the nationwide evolution in acute care hospitals, national cross-sectional surveys found a substantial decrease in the prevalence of methicillin resistant *Staphylococcus aureus* (MRSA) carriage in Belgian NHs (from 19.0% to 2005 to 9.0% in 2015), but at the same time an increase in the carriage of extended-spectrum β-lactamases-producing Enterobacteriaceae (from 6.2% to 2011 to 11.3% in 2015) [4–7].

Since 2009, Belgium monitors the burden of healthcare-associated infections (HAIs; i.e. infections acquired during a stay in a healthcare facility) and antimicrobial use in NHs by participating in the European point prevalence surveys (PPSs) of HAIs and antimicrobial use in LTCFs. These surveys, also known as HALT, are organised and funded by the European Centre for Disease Prevention and Control (ECDC). So far, three PPSs were conducted. In total, 722 LTCFs across 25 countries of the European Union (EU) and European Economic Area (EEA) participated in HALT-1 (2010). HALT-2 (2013) was organised in 1 181 LTCFs across 17 EU/EEA countries and HALT-3 (2016) took place in 3 052 LTCFs across 24 EU/EEA countries [8–11].

The current paper presents the main findings of the PPSs of HAIs and antimicrobial use in terms of prevalence and characteristics of the reported HAIs and antimicrobial prescriptions and available resources for infection prevention and control (IPC) and antimicrobial stewardship and this in (a) NHs participating in at least one of the surveys, and (b) a cohort of NHs that participated in all three consecutive surveys.

## Methods

### Study design and period

At ECDC’s invitation, the Belgian coordination centre, located at Sciensano (previously called the Scientific Institute of Public Health, Brussels), organised all three PPSs nationally. Each time, all Belgian NHs were invited to participate voluntarily. NH staff (e.g. coordinating physician, (head) nurse and/or quality coordinator) had to conduct the survey on one single day between 1 May and 30 September 2010 (HALT-1), between 1 April and 31 May 2013 (HALT-2) or between 1 September and 30 November 2016 (HALT-3). Detailed information about the ECDC’s study methodology is available elsewhere [2, 12, 13].

### Study documents and inclusion criteria

Ward lists collected information on the case mix (care load indicators and risk factors) of the total eligible population (i.e. all residents living full time in the facility since at least 24 h, present at 8:00 am on the day of the PPS and willing to participate in the survey). In contrast to the European methodology, this data was not aggregated but collected at individual resident level in HALT-2 and HALT-3. An institutional form collected aggregated case-mix data (HALT-1 only), and information on the organisation of medical care and the availability of IPC resources and antimicrobial stewardship elements. A resident questionnaire had to be completed for each eligible resident receiving an antimicrobial agent and/or presenting signs/symptoms of an active HAI on the PPS day [2, 12, 13].

Antimicrobial agents were grouped using the Anatomical Therapeutic Chemical (ATC) classification system of the World Health Organization Collaborating Centre for Drug Statistics Methodology [14].

Following agents for systemic use (oral, rectal, intravenous, intramuscular or inhalation treatments) were included: antibacterials (ATC level J01) and antimycotics (J02) for systemic use; antifungals (D01BA) for systemic use; antibiotics used as intestinal antiinfectives (A07AA); antiprotozoals (P01AB); antimycobacterials (J04) when used for treatment of mycobacteria including tuberculosis or as reserve treatment for multidrug-resistant bacteria. Antiviral agents, antiseptics and antimicrobial agents for topical use were excluded [2, 12, 13].

A HAI was considered active when signs/symptoms were present on the PPS day or if the signs/symptoms were present in the past but the resident was still receiving treatment for that infection on the survey day. The onset of symptoms had to occur more than 48 h after the resident was (re-)admitted to the LTCF. Moreover, symptoms had to be new or acutely worse and not related to a non-infectious cause [2, 12, 13]. In HALT-1, local surveyors had to tick signs/symptoms by infection site. During data analysis, the national coordinators applied a modified version of the McGeer criteria for infections in LTCFs on the reported signs/symptoms [12, 15]. In HALT-2 and HALT-3, decision algorithms were used in which local surveyors themselves had to tick signs/symptoms per infection site and apply modified case definitions of the Centers for Disease Control and Prevention (CDC) and the Society for Healthcare Epidemiology of America (SHEA) in order to confirm infections [2, 13, 16]. Since diagnostic confirmation tools (e.g. X-ray, microbiological results) are important criteria in the McGeer and CDC/SHEA case definitions, but not routinely available in European LTCFs, both criteria were somewhat altered to fit the European context [2, 12, 13]. For example, in HALT-2 and HALT-3 the definition of a UTI was adapted to include a ‘probable’ infection level for residents with UTI signs/symptoms but without microbiological confirmation, either because a urine culture test was not done or because the result was negative or not available in the facility [2, 13].

Data were collected using standardised paper forms and afterwards entered into the HALT stand-alone application by the local surveyors (HALT-1) or forwarded to the national coordinators for optical reading and analysis (HALT-2 and HALT-3).

### Statistical analyses

The prevalence of residents with at least one HAI or receiving at least one antimicrobial agent on the PPS day was defined as the total number of residents with at least one HAI or antimicrobial agent divided by the total number of eligible NH residents on the PPS day. Categorical variables were expressed as percentages, and continuous variables as median and interquartile range (IQR). We assessed the difference of NH characteristics across surveys using linear (for continuous variables) or logistic regression (for binary and binomial variables). We report the significance of overall differences across surveys (based on the F-test and likelihood-ratio chi-square test, respectively), and of the differences of HALT-2 and HALT-3 compared to HALT-1. All statistical analysis was performed using R Statistical Software version 4.1.0 (R Core Team, 2021).

### Post hoc analysis

We separately analysed data from 25 NHs that conducted all three PPSs to confirm whether changes in results (e.g. increase or decrease in prevalence or in percentage of available resources) were due to coincidence or were potentially influenced by different NHs participating in the different surveys.

## Results

### Participation

A total of 111 LTCFs participated in HALT-1: 107 NHs, two rehabilitation centres, one psychiatric facility and one mixed facility. In HALT-2, 87 NHs and one rehabilitation centre conducted the survey, while 158 NHs, four rehabilitation centres and three psychiatric facilities participated in HALT-3. Only the data of the participating NHs are presented here.

### Nursing home characteristics and available resources within the facilities

In all three surveys, more privately owned facilities participated. All but one (HALT-1 and HALT-2) and four (HALT-3) NHs indicated to have a designated physician in charge of coordinating the medical activities in the facility. In both HALT-1 and HALT-2, the most commonly reported tasks of these coordinating physicians were to clinically train nursing staff (85.9% and 91.9%, respectively), to coordinate the resident vaccination policy (79.3% and 86.0%, respectively) and to develop an IPC policy (70.8% and 72.1%, respectively) in the facility (data not collected in HALT-3).

The percentage of NHs having at least one person with IPC training available within the facility significantly increased between 2010 and 2016. In all three surveys, surveillance programs for resistant microorganisms were more commonly present within the facilities compared to surveillance programs for HAIs and antimicrobial use. Overall, antimicrobial stewardship elements were not commonly available within the NHs (Table 1). In 16.7% of the facilities antimicrobials are supplied by one pharmacy only, while 16.0% of the NHs work with one single microbiological laboratory only (HALT-3 only).

**Table 1:** Characteristics of nursing homes and residents participating in the Belgian HALT surveys, 2010-2016

	All NHs participating in at least one of the surveys				Cohort of NHs participating in all three consecutive surveys			
**NH characteristics**	**HALT-1 (2010)**	**HALT-2 (2013)**	**HALT-3 (2016)**	**p value**^a^	**HALT-1 (2010)**	**HALT-2 (2013)**	**HALT-3 (2016)**	***p*** **value**^a^
Participating facilities [N]	107	87	158		25	25	25	
Ownership [% public]	34.6	43.7	33.5	0.264	48.0	48.0	48.0	1.000
Rooms [median N, (IQR)]	97 (74-123)	94 (73-123)	100 (76-124)	0.603	101 (79-129)	99 (82-133)	99 (82-145)	0.925
Single rooms [%]	85.7	89.3 ***	92.2 ***	<0.001	88.7	90.6 *	90.2	0.069
Beds [median N, (IQR)]	103 (76-144)	100 (76-130)	106 (82-131)	0.592	110 (90-144)	101 (86.5-152)	103 (90-169)	0.941
***Infection prevention and control (IPC) resources within the facilities***								
Person with IPC training available [%]	50.5	65.5 *	71.3 ***	0.002	40.0	72.0 *	84.0 **	0.003
Help and expertise from an external IPC team (e.g. from a nearby hospital) on a formal basis [%]	77.1	77.6	80.3	0.806	79.2	83.3	84.0	0.894
Hand hygiene training in the previous year [%]	74.8	70.9	76.1	0.676	80.0	83.3	72.0	0.615
Surveillance program for HAIs [%]	43.0	39.8	39.7	0.852	44.0	58.3	60.0	0.461
Written protocol available in the NH for								
Management of MRSA and/or other MDRO [%]	98.1^b^	100.0	100.0	0.092	100.0	100.0	100.0	1.000
Hand hygiene [%]	99.1	97.6	100.0	0.122	100.0	100.0	100.0	1.000
Management of urinary catheters [%]	64.6	63.5	61.2	0.858	70.8	66.7	70.8	0.937
Management of venous catheters/lines [%]	37.8	31.7	30.0	0.464	36.4	36.4	34.8	0.992
Management of enteral feeding [%]	51.6	55.8	47.9	0.506	65.2	52.0	56.5	0.642
***Antimicrobial stewardship elements in place in the facilities***								
Restrictive list of antimicrobials to be prescribed [%]	10.5	14.5	16.1	0.418	8.0	16.7	16.0	0.587
Antimicrobial committee within the facility [%]	2.8	6.0	9.0	0.104	4.0	0.0	12.0	0.112
Annual training on appropriate antimicrobial prescribing provided [%]	11.2	9.5	12.3	0.812	16.0	12.5	20.0	0.775
A therapeutic formulary, comprising a list of antimicrobials [%]	62.6	70.2	52.3	0.019	80.0	83.3	68.0	0.412
Advice from a pharmacist for antimicrobials not included in the formulary [%]	15.0	20.2	22.6	0.297	24.0	16.7	20.0	0.815
Written therapeutic guidelines present in the NH for								
Respiratory tract infections [%]	35.5	56.9 **	49.5 *	0.018	60.0	68.4	78.9	0.399
Urinary tract infections [%]	32.7	59.6 **	54.0 **	<0.001	52.0	61.1	80.0	0.135
Wound and soft tissue infections [%]	43.9	68.4 **	45.4	0.006	56.0	77.8	65.0	0.324
Surveillance of antimicrobial consumption [%]	13.3	23.2	18.9	0.209	29.2	28.0	41.7	0.538
Surveillance of resistant microorganisms [%]	67.0	72.8	59.1	0.092	72.0	75.0	84.0	0.565
**NH population characteristics on survey day**								
Eligible residents [total N (median N; IQR]	11 911 (100; 74-140)	8 756 (93; 67-127)	16 215 (96; 75-122)	0.206	2 804 (106; 89-139)	2 748 (100; 87-136)	2 757 (100; 80-139)	0.977
Age ≥ 85 year [%]	52.9	60.3 ***	56.0 ***	<0.001	52.0	58.7 ***	54.7 *	<0.001
Gender [%]	25.6	24.9	25.5	0.486	27.1	26.7	27.6	0.758
Disoriented (in time and/or space) [%]	49.7	52.5 ***	55.0 ***	<0.001	55.3	56.2	58.1	0.095
Impaired mobility (wheelchair bound or bedridden) [%]	41.7	39.1 ***	38.3 ***	<0.001	45.6	42.4 *	40.9 ***	0.001
Incontinent for urine and/or faeces [%]	61.2	58.9 ***	55.9 ***	<0.001	66.0	61.7 **	63.4 *	0.004
Urinary catheter present [%]	2.4	2.8	3.1 ***	0.003	3.0	3.1	3.0	0.983
Vascular catheter present [%]	0.1	0.3 **	0.3 **	0.002	0.2	0.3	0.3	0.866
Pressure sore [%]	3.3	4.0 *	3.3	0.013	3.1	4.2 *	4.1 *	0.052
Wound other than pressure sore [%]	7.6	8.7 **	9.1 ***	<0.001	7.1	9.4 **	8.9 *	0.005
Surgery in the previous 30 days [%]	1.0	1.4 *	1.0	0.022	0.9	1.7 *	1.0	0.025

*NH *nursing home, *N *Number, *IQR* interquartile range, *IPC* infection prevention and control, *HAI* healthcare-associated infection, *MRSA* methicillin resistant *Staphylococcus aureus*, *MDRO *multidrug resistant microorganisms; ^a^ Regression analysis estimates significantly different from the reference (HALT-1) are indicated with asterisks. *: *p* ≤ 0.05, **: *p* ≤ 0.01, ***: *p* ≤ 0.001; ^b^ Management of MRSA only

### Characteristics of the eligible population

In all three surveys, approximately one in four residents were male. The median nursing care load was high with half or more of all residents being 85+ years, suffering from incontinence for urine and/or faeces and/or being disoriented in time and/or space. Around 40% of the eligible population was wheelchair bound or bedridden and approximately 10% had pressure sores or ‘other’ wounds such as ulcers, traumatic or surgical wounds or exit site wounds (e.g. from a gastrostomy or tracheostomy tube or from a suprapubic catheter). Urinary and vascular catheters were uncommon (Table 1).

### Healthcare-associated infections

**Fig. 1:**
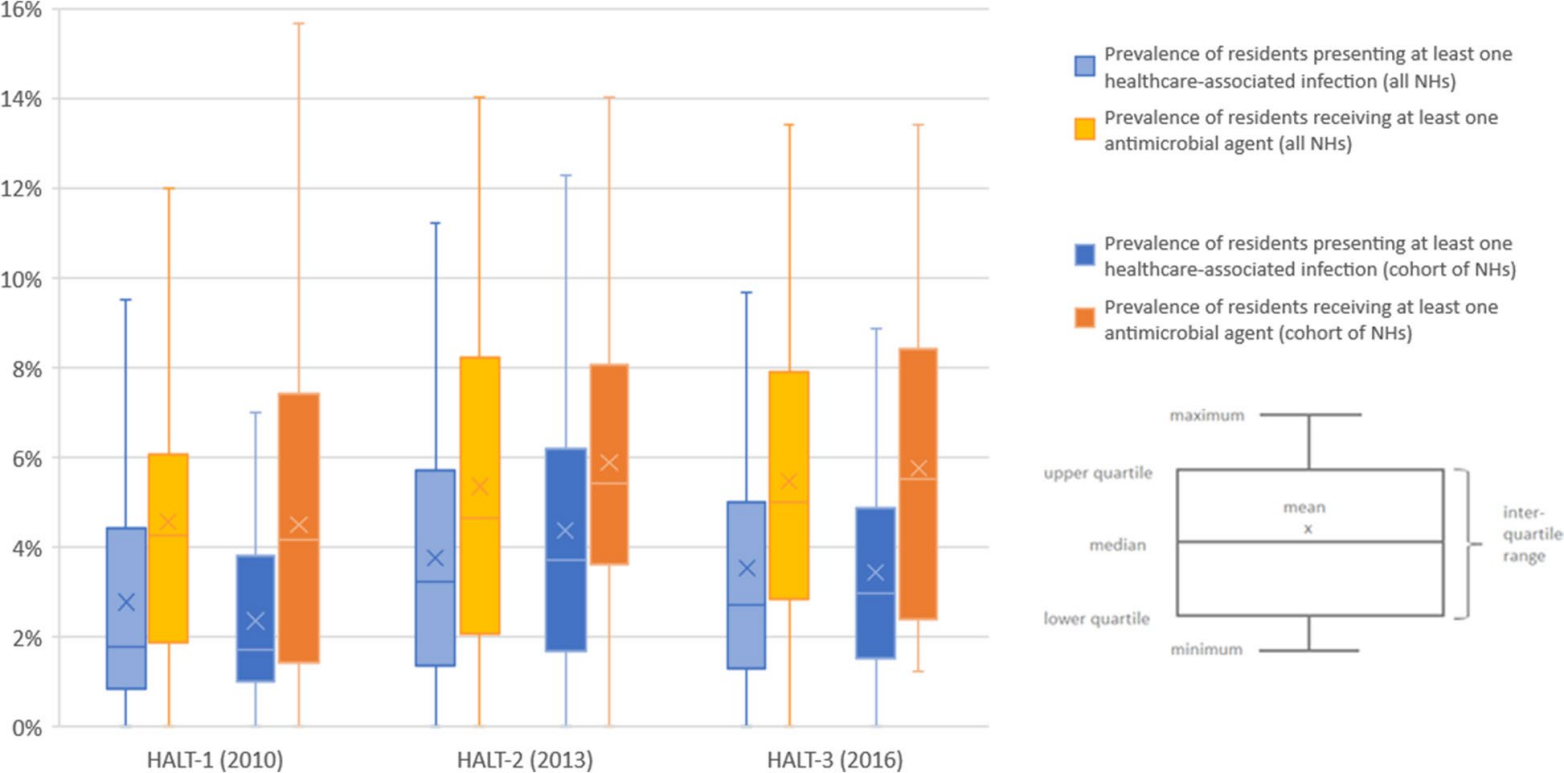
Prevalence of residents presenting healthcare-associated infections and/or receiving antimicrobial agents, Belgian HALT surveys, 2010-2016

In HALT-1, HALT-2 and HALT-3, 318 (2.7%), 314 (3.6%) and 547 (3.4%) residents presented one or more HAIs on the PPS day, respectively. The median age of these residents was 85 years (IQR: 79-90 years; 28.3% male) in HALT-1, 87 years (IQR: 82-91 years; 22.6% male) in HALT-2 and 87 years (IQR: 82-91; 25.1% male) in HALT-3. Figure 1 presents the median prevalence and spread of data by survey: 1.8% in HALT-1 (95% confidence interval (CI): 1.4-2.7%), 3.2% in HALT-2 (95% CI: 2.2-4.2%) and 2.7% in HALT-3 (95% CI: 2.1-3.4%).

A total of 344 infections were reported in HALT-1, 325 in HALT-2 and 557 in HALT-3. The distribution of HAIs by type of infection is shown in Table 2. In all three surveys, respiratory tract infections (RTIs) were most frequently reported, with lower RTIs representing more than 50% of this group. In HALT-1, skin or wound infections came in second place, while in HALT-2 and HALT-3 urinary tract infections (UTIs) preceded this infection group. For just over half of the residents with a UTI (51.4%) there were enough signs and/or symptoms and microbiological evidence to confirm the UTI.

**Table 2:** Number and prevalence of healthcare-associated infections reported in the Belgian HALT surveys, 2010-2016

	HALT-1 (2010)		HALT-2 (2013)		HALT-3 (2016)	
	n (%)	Crude prevalence (%)	n (%)	Crude prevalence (%)	n (%)	Crude prevalence (%)
**All HAI types**	**344 (100.0)**	**2.89**	**325 (100.0)**	**3.71**	**557 (100.0)**	**3.44**
**Respiratory tract infections (RTIs)**	**166 (48.3)**	**1.39**	**119 (36.6)**	**1.36**	**242 (43.4)**	**1.49**
Common cold/pharyngitis	57 (34.3)	0.48	35 (29.4)	0.40	106 (43.8)	0.65
Influenza-like illness	3 (1.8)	0.03	5 (4.2)	0.06	4 (1.7)	0.02
Pneumonia	15 (9.0)	0.13	10 (8.4)	0.11	11 (4.5)	0.07
Other lower RTIs	91 (54.8)	0.76	69 (58.0)	0.79	121 (50.0)	0.75
**Urinary tract infections (UTIs)**	**35 (10.2)**	**0.29**	**111 (34.1)**	**1.27**	**172 (30.9)**	**1.06**
Confirmed UTIs	-	-	57 (51.4)	0.65	100 (58.1)	0.62
Probable UTIs^a^	-	-	54 (48.6)	0.62	72 (41.9)	0.44
**Skin infections**	**67 (19.5)**	**0.56**	**45 (13.8)**	**0.51**	**88 (15.8)**	**0.54**
Cellulitis/soft tissue/wound infections	60 (89.6)	0.50	43 (95.6)	0.49	70 (79.5)	0.43
Herpes simplex or zoster infections	2 (3.0)	0.02	1 (2.2)	0.01	4 (4.5)	0.02
Fungal infections	5 (7.5)	0.04	1 (2.2)	0.01	14 (15.9)	0.09
Scabies	0 (0.0)	0.00	0 (0.0)	0.00	0 (0.0)	0.00
**Gastrointestinal infections**	**18 (5.2)**	**0.15**	**20 (6.2)**	**0.23**	**18 (3.2)**	**0.11**
Gastroenteritis	-	-	17 (85.0)	0.19	18 (100.0)	0.11
*Clostridioides difficile* infections	-	-	3 (15.0)	0.03	0 (0.0)	0.00
**Eye, ear, nose & mouth infections**	**34 (9.9)**	**0.29**	**15 (4.6)**	**0.17**	**18 (3.2)**	**0.11**
Conjunctivitis	24 (70.6)	0.20	10 (66.7)	0.11	12 (66.7)	0.07
Ear infections	2 (5.9)	0.02	3 (20.0)	0.03	0 (0.0)	0.00
Sinusitis	1 (2.9)	0.01	1 (6.7)	0.01	0 (0.0)	0.00
Oral candidiasis	7 (20.6)	0.06	1 (6.7)	0.01	6 (33.3)	0.04
**Bloodstream infections**	**1 (0.3)**	**0.01**	**1 (0.3)**	**0.01**	**0 (0.0)**	**0.00**
**Unexplained fever**	**2 (0.6)**	**0.02**	**9 (2.8)**	**0.10**	**8 (1.4)**	**0.05**
**Other infections**	**21 (6.1)**	**0.18**	**5 (1.5)**	**0.06**	**8 (1.4)**	**0.05**
**Unknown**	**-**	**-**	**-**	**-**	**3 (0.5)**	**0.02**

^a^ Sufficient signs/symptoms but no microbiological evidence (urine culture test not done or result negative or unknown) to confirm the UTI; HAI: healthcare-associated infections

### Antimicrobial use

On the PPS day, 514 (4.3%), 443 (5.1%) and 900 (5.6%) residents received one or more antimicrobials agents in HALT-1, HALT-2 and HALT-3, respectively. The median prevalence of residents using at least one antimicrobial agent ranged from 4.3% (95% CI: 3.5-4.8%) in HALT-1 to 4.7% (95% CI: 3.5-6.5%) in HALT-2 and 5.0% (95% CI: 4.2-5.9%) in HALT-3 (Fig. 1).

In total, 534, 455 and 928 antimicrobial agents were reported consecutively in three surveys. Almost all antimicrobials were administered orally: 96.8% in HALT-1, 98.2% in HALT-2 and 98.1% in HALT-3. A parenteral route (3.2% in HALT-1, 1.3% in HALT-2 and 1.5% in HALT-3) or other administration route (e.g. by inhalation; 0.4% in HALT-2 and HALT-3) were seldom used. The antimicrobial agents were most commonly prescribed in the NH: 91.0% in HALT-1 and HALT-2 and 88.0% in HALT-3. Prescriptions made in the hospital (8.0% in HALT-1, 8.1% in HALT-2 and 9.2% in HALT-3) or elsewhere (1.0% in HALT-1, 0.9% in HALT-2 and 2.8% in HALT-3) were rare.

**Fig. 2:**
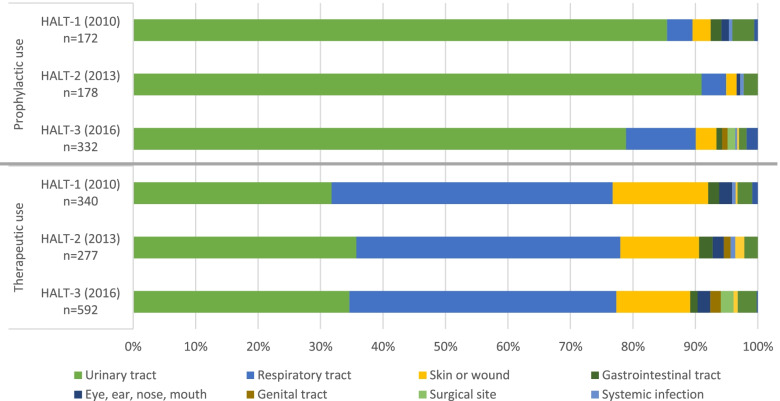
Indications for antimicrobial prescribing in nursing homes residents, Belgian HALT surveys, 2010-2016

Antimicrobials were most commonly prescribed for the therapeutic treatment of an infection: 66.4% in HALT-1, 60.9% in HALT-2 and 64.1% in HALT-3. In all three surveys, more than 85% of all therapeutic prescriptions were for RTIS, UTIs and skin or wounds infections (Fig. 2).

UTIs accounted for more than 75% of the total prophylactic use in all three surveys (Fig. 2). Of all antimicrobial agents recorded on the PPS day, 28.7% (HALT-1), 35.6% (HALT-2) and 28.4% (HALT-3) were prescribed for the prevention of a UTI (i.e. uroprophylaxis).

For 88.7% (*n* = 235/265; HALT-2) and 86.2% (*n* = 506/587; HALT-3) of the antimicrobials prescribed therapeutically, it was clearly stated in the resident’s medical or nursing record until when the antimicrobial should be given (end date) or when the treatment should be revised by the prescriber (review date). This end/review date was however only known for 3.4% (*n* = 6/178; HALT-2) and 8.5% (*n* = 28/330; HALT-3) of the prophylactic treatments.

The majority of the prescribed antimicrobial agents belonged to the J01 group of antibacterials for systemic use: 96.3% in HALT-1, 98.9% in HALT-2 and 94.6% in HALT-3. Table 3 presents the J01 subclasses prescribed for the therapeutic treatment of the three most common infections and overall. In all three surveys, ‘beta-lactam antibacterials, penicillins’, quinolones and ‘other antibacterials’ were most frequently prescribed for therapeutic use.

**Table 3:** Antibacterials for systemic use prescribed for therapeutic treatment, Belgian HALT surveys, 2010-2016

		J01A tetracyclines	J01B amphenicols	J01C beta-lactam antibacterials, penicillins	J01D other beta-lactam antibacterials	J01E sulfonamides and trimethoprim	J01F macrolides, lincosamides and streptogramins	J01G aminoglycoside antibacterials	J01M quinolone antibacterials	J0X other antibacterials	J01 antibacterials for systemic use Total
		n (%)	n (%)	n (%)	n (%)	n (%)	n (%)	n (%)	n (%)	n (%)	n (%)
HALT-1 (2010)	Respiratory tract infections	3 (2.0)	0 (0.0)	89 (58.6)	6 (4.0)	5 (3.3)	7 (4.6)	0 (0.0)	42 (27.6)	0 (0.0)	**152 (46.8)**
	Urinary tract infections	2 (1.9)	0 (0.0)	2 (1.9)	6 (5.6)	7 (6.5)	1 (0.9)	0 (0.0)	38 (35.2)	52 (48.2)	**108 (33.2)**
	Skin or wound infections	4 (9.1)	0 (0.0)	23 (52.3)	2 (4.6)	2 (4.6)	4 (9.1)	0 (0.0)	9 (20.5)	0 (0.0)	**44 (13.5)**
	**All infection groups**	**10 (3.1)**	**0 (0.0)**	**123 (37.9)**	**14 (4.3)**	**14 (4.3)**	**15 (4.6)**	**0 (0.0)**	**96 (29.5)**	**53 (16.3)**	**325 (100.0)**
HALT-2 (2013)	Respiratory tract infections	1 (0.9)	0 (0.0)	65 (56.0)	5 (4.3)	1 (0.9)	17 (14.7)	0 (0.0)	27 (23.3)	0 (0.0)	**116 (42.5)**
	Urinary tract infections	0 (0.0)	0 (0.0)	9 (9.2)	3 (3.1)	5 (5.1)	1 (1.0)	0 (0.0)	27 (27.6)	53 (54.1)	**98 (35.9)**
	Skin or wound infections	2 (5.7)	0 (0.0)	20 (57.1)	1 (2.9)	2 (5.7)	3 (8.6)	0 (0.0)	7 (20.0)	0 (0.0)	**35 (12.8)**
	**All infection groups**	**3 (1.1)**	**0 (0.0)**	**105 (38.5)**	**9 (3.3)**	**9 (3.3)**	**23 (8.4)**	**0 (0.0)**	**68 (24.9)**	**56 (20.5)**	**273 (100.0)**
HALT-3 (2016)	Respiratory tract infections	4 (1.6)	1 (0.4)	137 (55.5)	12 (4.9)	4 (1.6)	34 (13.8)	0 (0.0)	55 (22.3)	0 (0.0)	**247 (44.9)**
	Urinary tract infections	0 (0.0)	0 (0.0)	26 (12.8)	4 (2.0)	9 (4.4)	1 (0.5)	0 (0.0)	61 (29.9)	103 (50.5)	**204 (37.1)**
	Skin or wound infections	5 (9.1)	0 (0.0)	31 (56.4)	1 (1.8)	3 (5.5)	5 (10.9)	1 (1.8)	6 (10.9)	2 (3.6)	**55 (10.0)**
	**All infection groups**	**13 (2.4)**	**1 (0.2)**	**210 (38.2)**	**20 (3.6)**	**19 (3.5)**	**50 (9.1)**	**1 (0.2)**	**130 (23.6)**	**106 (19.3)**	**550 (100.0)**

Antimicrobials presented according to the Anatomical Therapeutic Chemical (ATC) classification system level 3 subclasses; no J01R combinations were reported

‘Other antibacterials’ represented 83.3% (*n* = 140/168), 91.0% (*n* = 161/177) and 77.2% (*n* = 250/324) of all J01 antibacterials prescribed for prophylactic use.

### Results of the cohort of nursing homes that participated in all three surveys

A description of the 25 NHs and their eligible population participating in all three PPS can be found in Table 1. In HALT-1, HALT-2 and HALT-3, 67 (2.4%), 118 (4.3%) and 88 (3.2%) residents presented one or more HAI, while 128 (4.6%), 158 (5.7%) and 159 (5.8%) residents used one or more antimicrobials agents on the survey day, respectively. The median prevalence of residents with HAI ranged from 1.7% (95% CI: 1.2-2.4%) in HALT-1 to 3.7% (95% CI: 2.3-5.5%) in HALT-2 and 3.0% (95% CI: 1.7-4.6%) in HALT-3. The median prevalence of residents using at least one antimicrobial agent was 4.2% (95% CI: 1.8-5.9%) in HALT-1, 5.4% (95% CI: 3.9-7.5%) in HALT-2 and 5.5% (95% CI: 2.6-7.9%) in HALT-3 (Fig. 1). The characteristics of the HAIs and the prescribed antimicrobial agents are presented in Additional file 1.

## Discussion

In this paper we report the results of three PPSs of HAI and antimicrobial use consecutively (2010, 2013 and 2016) conducted in Belgian nursing homes. The median prevalence of residents receiving at least one antimicrobial agents increased from 4.3% in HALT-1 to 4.7% in HALT-2 and 5.0% in HALT-3. The median prevalence of residents presenting at least one HAI on the survey date varied from 1.8% in HALT-1 to 3.2% in HALT-2 and 2.7% in HALT-3. Based on the HALT-2 point prevalence estimates, the total number of HAIs, with an average duration of 10 days, in Belgian NHs (more than 1 500 facilities with around 136 000 beds) was estimated at 170 090 infections annually [9].

By participating in this European study and applying a standardised methodology, Belgium is able to compare its prevalence rate and other figures with other EU/EEA Member States. In all three surveys, our prevalence rates were (slightly) higher than the European prevalence and this for both HAIs (median EU/EEA prevalence: 1.5% in HALT-1, 2.8% in HALT-2 and 2.1% in HALT-3) and antimicrobial use (median EU/EEA prevalence: 3.4% in HALT-1 and 3.6% in HALT-2 and HALT-3) [8–11]. Although the data were collected in a similar way and during the same time period, results should still be interpreted carefully. First of all, the number of participating LTCFs and eligible residents varied significantly between countries. Moreover, a NH in Belgium might not be comparable to a NH in another EU Member State. The resident population can vary because of cultural differences between countries and the type of care provided in these facilities is dependent on the other services and facilities available within the healthcare system [17]. Belgium makes a distinction between two levels of long-term care for the elderly. The lowest level provides care in a home-replacing environment when possibilities for home care or short-term residential care are not sufficient anymore. The highest level is intended for older adults who are highly dependent on the help of others for their activities of daily living. Both levels of care can, but not necessarily have to be, provided in the same facility [18]. The NHs that participated in this survey provided both levels of long-term care for the elderly. By Royal Decree, these facilities should have a CP, i.e. a general practitioner (GP) who takes on the medical coordination in the facility [19]. This is a challenging task as on average 35 personal GPs per 100 residents are visiting our NHs to provide medical care to the residents [20]. For this reason, antimicrobial stewardship elements are more difficult to put in place and were consequently less frequently reported than IPC resources in our survey. Only the percentage of NHs reporting to have a person with IPC training (e.g. doctor or (head)nurse) available within the facility significantly increased between 2010 and 2016, a finding confirmed by our post hoc analysis. There are currently no legal minimal requirements for having a person with IPC training in Belgian NHs, but the CP should develop a policy for the control of HAIs in collaboration with the head nurse(s) [19].

The median prevalence of residents with at least one antimicrobial agent non-significantly increased between HALT-1 and HALT-3. Our post-hoc analysis (including NHs participating in all three surveys) found a similar non-significant increase in the prevalence. Prior to HALT, two surveys were conducted in 2009 in light of the European Surveillance of Antimicrobial Consumption (ESAC) NH subproject using a comparable methodology to measure antimicrobial use. These studies found a median prevalence of 5.1% in April 2009 (116 Belgian NHs; 12 085 eligible residents) and 4.4% in November 2009 (103 Belgian NHs; 11 160 eligible residents). In both the ESAC-NH and HALT surveys, ‘other antibacterials’, ‘beta-lactam antibacterials, penicillins’ and ‘quinolone antibacterials’ were the most frequently prescribed subclasses of antibacterials for systemic use [21, 22].

At present, there are no nationally accepted guidelines for antimicrobial use in an elderly population in general or for treatment of infections in NHs in particular. The HALT surveys though highlight the need to establish guidelines for the treatment and prevention of UTIs, as up to more than 50% of all antimicrobials reported in these PPSs were used for an indication related to the urinary tract. Moreover, uroprophylaxis accounted for more than 28% of the total antimicrobial use. In HALT-2, this rate was even higher (35.6%). This difference can however partially be explained by the emphasis that was put on UTIs during training and by additional questions on UTIs in the HALT-2 questionnaires (optional UTI module at EU/EEA level; data not presented here) [9, 13, 23]. The additional workload associated with this UTI module might also partially explain the lower participation rate in 2013.

More in-depth research is needed to explore why general practitioners prescribe that much prophylactic treatments. At present, long-term antimicrobial therapy for the prevention of UTIs is not recommended in an elderly LTCF population as its efficacy has not yet been documented in appropriate trials [24]. In this paper, we however noted that for less than 10% of all prophylactic prescriptions an end/review date was recorded in the resident’s medical or nursing file.

Our primary and post-hoc analyses both showed a significant increase in the median prevalence of residents with at least one HAI between HALT-1 and HALT-2 and a non-significant decrease between HALT-2 and HALT-3. In HALT-2 and HALT-3, RTIs were the most commonly reported infections, followed by UTIs and skin infections. The same infection groups were also most frequently reported in 2010, but the proportions were somewhat different, i.e. RTIs followed by skin or wound infections and then UTIs. The results of the two most recent PPSs are however difficult to compare with those of the 2010 PPS as a different methodology was used to collect the HAI data. In HALT-2 and HALT-3, data on HAI were collected by asking surveyors to apply decision algorithms based on the CDC/SHEA surveillance definitions of infections in LTCFs [2, 13, 16]. In HALT-1, a modified version (i.e. with addition of the criterion ‘diagnosis by the attending physician’) of the McGeer criteria for surveillance of infections in LTCFs was used [12, 15]. Moreover, in HALT-1 local surveyors did not have to apply the definitions themselves, but only had to report the signs/symptoms present on the day of the survey. The definitions were only applied upon data analysis.

The HALT-3 methodology for collecting HAIs originally also included HAIs associated to other healthcare facilities (e.g. hospitals or other LTCFs) [2]. We deliberately did not report on these HAIs in order to have prevalence rates comparable to the previous studies. Besides the 557 HAIs associated to the NH itself and reported in this paper, there were also 35 HAIs associated to other healthcare facilities and 130 HAIs with an unknown or missing origin of infection [25]. This high percentages of missing data on HAI origin (18.0%) demonstrates that the methodology was most likely too difficult for NH staff members to apply and could potentially explain why the HAI prevalence in HALT-3 was lower compared to HALT-2 using the same set of infection definitions.

The two previously described major changes in HAI data collection methodology (i.e. a change in applied HAI definitions in between HALT-1 and HALT-2 and the inclusion of infections also acquired in other healthcare facilities in HALT-3) concerned the European protocol. In combination with seasonal variation, these presumably explain why we can observe a similar HAI prevalence trend at both national and EU level, with the highest HAI prevalence rates reported during HALT-2. Our surveys had several strengths and weaknesses. None withstanding PPSs provide only a snapshot of a situation at a particular point in time, the surveys provided valuable insight in the burden of HAIs and antimicrobial use in Belgian NHs. All Belgian NHs were invited to voluntarily participate in this survey. No financial incentives were given. Although selecting a random sample of NHs would have been better for the generalisability of the results, we found it important to give all NHs the possibility to participate. By conducting the survey themselves, local NH staffs learn how and why to monitor infections and antimicrobial use in their own facility. Non-mandatory training sessions were organised to ensure local surveyors understand the principles of surveillance, to familiarize them with the study’s methodology and tools and to enable nursing staff to understand their results and identify key areas for improvement.

Because of the low participation rate (5-10% of all invited facilities) the data cannot be considered to be representative for Belgium. It is likely that NHs with more IPC resources agreed to participate. No data are available to compare participants to non-participants and therefore confirm this hypothesis.

The prevalence of residents with HAIs or antimicrobial use presented here might be an underestimation. We did not conduct a PPS during the winter, when a peak in infections, and therefore also in antimicrobial use, can be expected. The surveys were conducted at different moments of the year, so seasonal variation in the prevalence of HAIs cannot be excluded. Infections were also reported by NH personnel who is not familiar with the use of surveillance criteria. Moreover, antimicrobial use data were not always collected or validated by a physician. Epstein et al. compared data collected by NH personnel for HAI surveillance with data collected by trained surveillance officers. They found discrepancies in the estimates of HAI prevalence between both groups which were likely attributable to differences in the level of familiarity with surveillance and in the methods of data collection. Despite having access to a larger variety of data sources including direct observations and verbal reports for caretakers, NH personnel identified fewer residents with any HAI screening criteria than the surveillance team. They more often missed antimicrobial use as an HAI screening criterion [26]. Although recommended, we did not impose direct observation of residents to our participating NHs. Therefore, mild infection episodes might have been missed by the local surveyors especially if no antimicrobial was prescribed. Because of ethical restrictions (i.e. difficulties in obtaining permission to review the residents’ medical files), we did not conduct a validation study to assess the sensitivity and specificity of the data collected. Within the framework of the HALT-2 survey, 10 other countries however did organise a validation study. The specificity for both HAIs and antimicrobials use was 99%. The sensitivity was lowest for HAIs (76%), and highest for antimicrobial use (90%) [9].

## Conclusions

None withstanding the limitations peculiar to the study design, the study provided a good opportunity to enhance surveillance skills at local level and to collect valuable information on and increase awareness for HAIs and antimicrobial use in a frail older NH population at both national and local level.

## Supplementary Information

**Additional file 1. **Characteristics of healthcare-associated infections and antimicrobial prescriptions as reported by the 25 nursing homes participating in all three Belgian HALT surveys, 2010-2016. Table presenting the characteristics of healthcare-associated infections and antimicrobial prescriptions as reported by the 25 nursing homes participating in all three Belgian HALT surveys, 2010-2016.

## Data Availability

The datasets used and/or analysed during the current study are available from the corresponding author on reasonable request.

## Abbreviations

ATC: anatomical therapeutic chemical
CDC: Centers for Disease Control and Prevention
CI: confidence interval
CP: coordinating physician
ECDC: European Centre for Disease Prevention and Control
EEA: European Economic Area
ESAC: European surveillance of antimicrobial consumption
EU: European Union
GP: general practitioner
HAI: healthcare-associated infection
HALT: healthcare-associated infections and antimicrobial use in European long-term care facilities
IPC: infection prevention and control
LTCF: long-term care facility
MDRO: multidrug-resistant organism
MRSA: methicillin resistant *Staphylococcus aureus*
NH: nursing home
PPS: point prevalence survey
RTI: respiratory tract infection
SHEA: Society for Healthcare Epidemiology of America
UTI: urinary tract infection
