# Supplementary material for: Healthcare-associated infections and antimicrobial use in Belgian nursing homes: results of three point prevalence surveys between 2010 and 2016
Source: Arch Public Health. 2022 Feb 18;80:58. doi: 10.1186/s13690-022-00818-1 (PMC8855602; doi:10.1186/s13690-022-00818-1)
Supplement: Supplementary file 1 — Additional file 1. Characteristics of healthcare-associated infections and antimicrobial prescriptions as reported by the 25 nursing homes participating in all three Belgian HALT surveys, 2010-2016. Table presenting the characteristics of healthcare-associated infections and antimicrobial prescriptions as reported by the 25 nursing homes participating in all three Belgian HALT surveys, 2010-2016. [file 13690_2022_818_MOESM1_ESM.docx]

**Additional file 1** Characteristics of healthcare-associated infections and antimicrobial prescriptions as reported by the 25 nursing homes participating in all three Belgian HALT surveys, 2010-2016

|  | **HALT-1 (2010)** | **HALT-2 (2013)** | **HALT-3 (2016)** |
| --- | --- | --- | --- |
|  | **n (%)** | **n (%)** | **n (%)** |
| **Healthcare-associated infections (HAIs)** | | | |
| Urinary tract infections (UTIs) | 8 (11.4) | 31 (25.8)^1^ | 34 (37.4)^2^ |
| Respiratory tract infections (RTIs) | 34 (48.6) | 54 (45.0) | 40 (44.0) |
| Skin infections | 17 (24.3) | 17 (14.2) | 11 (12.1) |
| **All HAIs** | **70 (100.0)** | **120 (100.0)** | **91 (100.0)** |
| **Antimicrobial prescriptions** | | | |
| ***Indications for antimicrobial use*** | | | |
| Prophylactic prescriptions for UTIs | 33 (82.5) | 61 (87.1) | 39 (68.4) |
| **All prophylactic prescriptions** | **40 (31.8)**^3^ | **70 (43.2)**^3^ | **57 (35.2)**^3^ |
| Therapeutic prescriptions for UTIs | 27 (31.4) | 25 (27.2) | 41 (39.1) |
| Therapeutic prescriptions for RTIs | 42 (48.8) | 49 (53.3) | 42 (40.0) |
| Therapeutic prescriptions for skin or wound infections | 13 (15.1) | 11 (12.0) | 13 (12.4) |
| **All therapeutic prescriptions** | **86 (68.3)**^3^ | **92 (56.8)**^3^ | **105 (64.8)**^3^ |
| ***End/revision date noted in the resident’s medical or nursing record***^4^ | | | |
| Date known for prophylactic prescriptions | - | 4/70 (5.7) | 8/56 (14.3) |
| Date known for therapeutic prescriptions | - | 78/88 (88.6) | 89/104 (85.6) |
| ***ATC J01 antibacterials for systemic use prescribed (therapeutic use only)*** | | | |
| J01A tetracyclines | 4 (4.9) | 0 (0.0) | 1 (1.0) |
| J01C beta-lactam antibacterials, penicillins | 33 (40.2) | 33 (36.7) | 35 (35.0) |
| J01D other beta-lactam antibacterials | 3 (3.7) | 3 (3.3) | 5 (5.0) |
| J01E sulfonamides and trimethoprim | 3 (3.7) | 4 (4.4) | 4 (4.0) |
| J01F macrolides, lincosamides and streptogramins | 2 (2.4) | 8 (8.9) | 12 (12.0) |
| J01M quinolone antibacterials | 18 (22.0) | 29 (32.2) | 20 (20.0) |
| J0X other antibacterials | 19 (23.2) | 13 (14.4) | 23 (23.0) |
| **All J01 antibacterials for systemic use** | **82 (95.4)**^5^ | **90 (97.8)**^5^ | **100 (95.2)**^5^ |

^1^ 48.4% confirmed UTIs (i.e. sufficient signs/symptoms but no microbiological evidence (urine culture not done, negative or unknown)); ^2^ 79.4% confirmed UTIs; ^3^ % of all antimicrobial prescriptions; ^4^ it is clearly stated in the resident’s medical or nursing record until when the antimicrobial should be given (end date) or when the antimicrobial treatment should be revised by the prescriber (review date); ^5^ % of all antimicrobials prescribed for therapeutic use; antimicrobials presented according to the Anatomical Therapeutic Chemical (ATC) classification system level 3 subclasses; no J01B amphenicols or J01G aminoglycoside antibacterials prescribed.
